# Association between serum vitamin D levels and lipid profiles: a cross-sectional analysis

**DOI:** 10.1038/s41598-023-47872-5

**Published:** 2023-11-29

**Authors:** Amir Gholamzad, Niloofar Khakpour, Tina Kabipour, Mehrdad Gholamzad

**Affiliations:** 1 Department of Laboratory Medicine, Faculty of Paramedical Sciences, Tehran Medical Sciences, Islamic Azad University, Tehran, Iran; 2 Farhikhtegan Medical Convergence Sciences Research Center, Farhikhtegan Hospital, Tehran Medical Sciences, Islamic Azad University, Tehran, Iran; 3 Department of Bacteriology and Virology, Shiraz University of Medical Sciences, Shiraz, Iran; 4 Department of Microbiology and Immunology, Faculty of Medicine, Tehran Medical Sciences, Islamic Azad University, Tehran, Iran

**Keywords:** Biochemistry, Biological techniques, Biomarkers, Diseases, Endocrinology, Medical research

## Abstract

Vitamin D is an essential nutrient that plays a crucial role in calcium homeostasis and bone health. Recent research suggests that vitamin D may also have an impact on lipid metabolism, specifically the level of circulating lipids in the blood. We aim to investigate it role among healthy participate. We conducted a cross-sectional study of 15,600 patients who were referred to the laboratories of university hospitals. We measured the serum levels of Vitamin D as well as triglycerides, total cholesterol, LDL, and HDL using ELISA. We found that the mean serum level of Vitamin D was 40.31 ± 20.79 ng/mL. Of the participants, 16.7% had a serum level of Vitamin D less than 20 ng/mL, 57.7% had a level between 21 and 40 ng/mL, and 13.5% had a level between 41 and 60 ng/mL. Additionally, 12.2% had a level greater than 60 ng/mL. We performed a one-way analysis of variance and found that as the serum level of Vitamin D increased, the mean LDL level decreased significantly. Our study provides evidence of a significant relationship between serum levels of Vitamin D and LDL levels in patients. The findings suggest that vitamin D status may play a role in regulating lipid metabolism and may have implications for the prevention and treatment of cardiovascular disease. Further research is needed to elucidate the underlying mechanisms of this relationship and to determine optimal levels of vitamin D intake for maintaining lipid profiles.

## Introduction

Recently, due to the multiple effects of vitamin D on health, its status in different individuals has become a growing research topic^1^. The classic effects of vitamin D are mediated by its active metabolite, 1,25-dihydroxy vitamin D, which enables the absorption of calcium in the intestines, maintenance of adequate phosphate levels for bone mineralization, bone growth, and remodeling^2^. The biological effects of vitamin D are regulated by vitamin D receptors present in other tissues that are not related to calcium metabolism^3^.

Various studies have shown that vitamin D can be stored in adipose tissue^4^. Adipose tissue has the potential to accumulate a significant amount of vitamin D, particularly when fat mass expands(such as in overweight and obesity)^5^. It has been demonstrated that obese individuals have lower circulating concentrations of (OH)D25 compared to non-obese individuals. However, it can be said that body fat mass and percentage of body fat are strong predictors of vitamin D status in different individuals^6^.

Initial studies proposed a hypothetical mechanism suggesting that increased vitamin D in circulation with obesity decreases hepatic synthesis of (OH)D25 through a negative feedback mechanism, leading to a decrease in serum (OH)D25^7^. Assessing vitamin D status is reflected by measuring its metabolite in circulation compared to the active form. However, the prevalence of deficiency or sufficiency of serum (OH)D25 varies greatly depending on the criteria used to define it, including population, season, dietary habits, ethnicity, physical activity, and age range^8^.

In general, dyslipidemia refers to an imbalance in the levels of blood lipids, including triglycerides (TG), total cholesterol (TC), low-density lipoprotein cholesterol (LDL-c), and high-density lipoprotein cholesterol (HDL-c)^9^. This disorder is recognized as a risk factor for the development of atherosclerosis-related diseases such as coronary heart disease, ischemic cerebrovascular disease, and peripheral vascular disease^10^. Various factors, such as aging, increased intake of fats, especially saturated and trans fats, and decreased intake of antioxidant-rich foods like fruits and vegetables, play a role in its etiology^11^.

Vitamin D has multiple roles in addition to its role in regulating calcium homeostasis and contributes to maintaining human health. Recent years have seen an upward trend in vitamin D deficiency. The primary source of vitamin D is sunlight exposure, and certain protein-rich foods also contain vitamin D. However, it should be noted that dietary intake alone may not meet individuals' vitamin D needs. Observational studies have shown that low serum vitamin D levels are an independent risk factor for hypertension and cardiovascular diseases^12^.

Vitamin D is a fat-soluble hormone that is naturally synthesized in the body through subcutaneous synthesis upon exposure to sunlight. This vitamin plays a crucial role in maintaining the health of bones, muscles, and also prevents various diseases such as cancer, diabetes, cardiovascular diseases, and autoimmune diseases^13^. Based on available evidence, vitamin D deficiency is directly associated with mortality in patients with cardiovascular diseases (CVD), and improving the vitamin D status has been reported to reduce the risk of myocardial infarction and stroke through multiple studies^14^. Dyslipidemia is one of the major risk factors for developing CVD, and significant associations have been found between serum vitamin D levels and lipid profiles according to conducted studies^15^. Although multiple mechanisms have been proposed to explain the effects of vitamin D on lipid profiles, the impact of this vitamin on blood lipid levels is still not clear^16^. Proposed mechanisms suggest that vitamin D may directly affect serum lipid profiles, including triglycerides, total cholesterol, and LDL cholesterol, by increasing the production of bile salts and reducing the activity of lecithin-cholesterol acyltransferase, as well as indirectly through its influence on calcium absorption, resulting in decreased fat absorption and increased synthesis of hepatic bile acids from cholesterol^17^. Considering the vital role mentioned for serum vitamin D levels, it can be inferred that improving the serum vitamin D level may have a significant role in improving associated conditions such as hyperlipidemia in affected individuals. Therefore, we aim to investigate the relationship between vitamin D levels and blood lipid levels in this study.

## Patients

We confirm that all methods were carried out in accordance with relevant guidelines and regulations, and that all experimental protocols were approved by the Institutional Review Board (IRB) of the Tehran Islamic Azad University of Medical Sciences. Informed consent was obtained from all subjects and/or their legal guardian(s).

In this research data from healthy patients were collected and we confirm that Cardiovascular diseases and metabolic diseases such as liver and kidney diseases, as well as autoimmune patients and patients using vitamins, were excluded from this study.

## Methods

This is a descriptive, cross-sectional study conducted on people who visited the laboratories of Tehran between January 2020 and January 2023. The calculated sample size for this research was 15,600 individuals which were suitable for the propos of the research.

In this analytical cross-sectional study, 15,600 patients who visited the laboratories of Tehran were selected and examined based on the results of available ELISA and Biochemical tests to determine the presence or absence of hyperlipidemia. Demographic information such as age and gender were recorded in a prepared checklist that contained the investigated variables. The patients were also evaluated for their serum vitamin D levels. The level of vitamin D was compared to the levels of total cholesterol, LDL, HDL, and triglycerides, and a correlation was found between these variables. Additionally, other variables were compared as well.

### Laboratory methods

Samples from these individuals were collected during morning hours while they were in a fasting state. Additionally, during this collection, participants were asked about their vitamin D intake and underlying diseases as background information. Serum samples from patients were separated using centrifugation, and an ELISA test was performed to measure the levels.

### Data analysis method

All data were statistically analyzed using SPSS version 26 and ANOVA. Mean and standard deviation were calculated for quantitative variables, while absolute and relative frequency were calculated for qualitative variables. The hypotheses of the research were tested using the t-test of two independent samples. In this study, we set a P-Value less than 0.05 as the basis for determining the significance of our results.

### Ethical approval

All methods were carried out in accordance with relevant guidelines and regulations, and that all experimental protocols were approved by the Institutional Review Board (IRB) of the Tehran Islamic Azad University of Medical Sciences.

## Results

In this study, a total of 15,600 people who visited the laboratory of Islamic Azad hospitals in Tehran between January 2020 and January 2023 were included. The demographic information of the participants was checked, and the research hypotheses were tested, leading to the following results.

Based on the results in the Table 1, 41% of the study participants were male and 59% were female, and the average age of the patients is 43–79 years.

**Table 1:** Frequency distribution and distribution of age and gender of study participants.

Gender	Prevalence	Average
Male	6400 (41%)	46/80
Female	9200 (59%)	41/69
Total	15,600	44.24

Based on the results of the Table 2, the average serum level of Vitamin D has been 40.31 ng/ml ± 20.79, the serum level of Vitamin D was less than 20 in 16.7% of people, 21–40 in 57.7% of people, and 41–6 in 13.5% of people; Also, the serum level of Vitamin D was more than 60 ng/ml in 12.2% of people and no toxic level of Vitamin D were observed.

**Table 2:** Frequency distribution of serum level of Vitamin D.

Level of vitamin D	Abundance	Result interpretation
Less than 20	2600 (16.7%)	Insufficient or a potential deficiency
21–40	9000 (57.6%)	Normal
41–60	2100 (13.5%)	Sufficient
More than 60	1900 (12.2%)	High

Based on the result of Table 3 the average triglyceride level of the study participants was 183.39 ± 89.51 mg/dL. Also, triglyceride level was 200 or more in 30.8% of people participating in the study.

**Table 3:** Serum Levels of Triglyceride.

Level of triglyceride	Abundance	Result interpretation
Less than 150	5800 (37.2%)	Normal
150–199	5000 (32.1%)	Borderline high
More than 200	4800 (30.7%)	High

Based on Table 4 results, the average total cholesterol level of the study participants was 189.25 ± 38.96 mg/dL; Also, total cholesterol level was 240 or more in 10.9% of people participating in the study.

**Table 4:** Frequency distribution of Serum Levels of Cholesterol.

Level of cholesterol	Abundance	Result interpretation
Less than 200	10,200 (65.4%)	Normal
200–239	3700 (23.7%)	Borderline high
More than 240	1700 (10.9%)	High

Based on Table 5 the average HDL level of the study participants was 48.87 and the LDL level was 86.64 mg/dL.

**Table 5:** HDL and LDL levels distribution.

Average	Lipid profile
48/87	HDL
86/64	LDL

Based on the data presented in Table 6 and the results of the K-2 test, it can be concluded that there is no statistically significant association between the serum level of Vitamin D and gender.

**Table 6:** Relationship ﻿between the levels of Vitamin D and gender.

Level of vitamin D	Gender		Result interpretation	*P*-Value	Q2
	Male	Female			
Less than 20	600 (23.1)	2000 (76.9)	Insufficient or a potential deficiency	0.051	7.78
21–40	4200 (46.7)	4800( 53.3)	Normal		
41–60	11,000 (52.4)	1000(47.6)	sufficient		
More than 60	500 (26.3)	1400(73.7)	High		

According to the findings presented in Table 7 and the results of the one-way analysis of variance test, a significant correlation exists between the serum level of Vitamin D and age. Specifically, the average serum level of Vitamin D increases significantly with age (with a P-value less than 0.05).

**Table 7:** The distribution of Vitamin D levels and age.

Age	Average	*P*-Value	F-test
Less than 18	26.54	< 0.05	9
18–45	29.63		
45–60	43.32		
More than 60	49.95		

According to Table 8, there were no significate correlations between Vitamin D and Total Cholesterol, Triglyceride and HDL but an Inverse correlation between LDL and Vitamin D was observed.

**Table 8:** Determining the relationship between the levels of Vitamin D and Blood Lipids.

Level of vitamin D	Less than 20	21–40	41–60	More than 60	*P*-Value	F-Test
Total Cholesterol	178.73	189.19	191.19	201.84	> 0.05	3.11
Triglyceride	149.15	188.81	189.90	197.42	> 0.05	1.56
HDL	56.2	58.1	61.2	62.1	> 0.05	7.45
LDL	111.7	110.6	106	105.09	< 0.05	6.45

## Discussion

Epidemiological studies show a reverse association between the level of Vitamin D (25(OH)D) and cardiovascular risk biomarkers, including atherogenic lipid profiles. Vitamin D deficiency is very common and can be effectively treated through dietary supplementation. However, the role of supplementation in modifying cardiovascular risks is not well-established, and it is unclear whether vitamin D status is causally related to the disease or simply a health indicator. This is of importance to physicians and the general population due to the increasing use of over-the-counter vitamin D supplements. Vitamin D deficiency is highly prevalent and is associated with dyslipidemia and cardiovascular diseases. The impact of correcting vitamin D deficiency on blood lipids and cardiovascular risk factors remains unknown. This study aimed to investigate the level of Vitamin D on blood lipid profiles, which are predictive factors for cardiovascular diseases. For this purpose, 15,600 individuals with an average age of 43.79 years visiting Tehran's Islamic Azad university hospitals were examined.

According to the study results, the mean serum level of Vitamin D was 20.79 ± 31.40 ng/ml^18^. The serum level of Vitamin D was less than 20 ng/mL in 16.7% of individuals, 21–40 ng/mL in 57.13% of individuals, and 41–60 ng/mL in 13.5% of individuals. Additionally, the serum level of Vitamin D was above 60 ng/mL in 2.12% of individuals^20^. In the study by Faridi et al., it was also demonstrated that 15% of individuals had vitamin D deficiency, which was relevant to the current study^18^. Furthermore, in the study by Kumaratne et al., vitamin D levels were estimated to be below normal in 20% of individuals^19^. In the study conducted by Khoo et al., the vitamin D level was estimated to be below 20 ng/mL in 17% of individuals, which was within the scope of the present study^19^. Upon reviewing foreign literature, it was found that the level of Vitamin D in Iran did not differ significantly from other countries^18^.

Furthermore, there was no significant difference between the serum levels of Vitamin D and total cholesterol in men and women. In a study conducted by Asmi et al., it was shown that there was no observed relationship between changes in blood cholesterol and vitamin D levels when stratified by gender, which is consistent with the current study^21^. Additionally, in a study by Abdi et al., it was demonstrated that there is a significant relationship between serum vitamin D levels and total cholesterol in women, which is not applicable to the present study^22^.

According to the results of this investigation, no significant relationship was observed between serum vitamin D levels and triglycerides, HDL, and total cholesterol. However, an increase in serum vitamin D levels was associated with a decrease in LDL. This finding is inconsistent with the study by Ponda et al., which demonstrated that an increase in vitamin D levels leads to an increase in HDL^23^. In the study by Kumaratne et al., high vitamin D levels were associated with a decrease in LDL, which is in line with the present study^19^. Additionally, the study by Asmi et al. showed that LDL levels were lower in individuals without vitamin D deficiency compared to those with vitamin D deficiency^21^. On the contrary, the study by Kim et al. indicated that there was no significant relationship between vitamin D levels and cholesterol, LDL, and HDL, and triglyceride levels were significantly higher in individuals with vitamin D deficiency, which is not consistent with the findings of the present study^24^. Furthermore, in the study conducted by Rossouw et al., no significant relationship was observed between changes in blood lipids and vitamin D levels, which contradicts the findings of the current study^25^. In the study by Keshvari et al., no significant relationship was found between vitamin D levels and triglycerides, which aligns with the present study^26^.

Epidemiological evidence, primarily based on relevant studies, suggests a role for higher levels of Vitamin D in protecting against cardiovascular diseases. However, this level of evidence does not establish a causal relationship, and until convincing evidence is available to inform clinical practice, cross-sectional analyses of large laboratory databases provide a valuable tool for addressing unresolved health questions, although longitudinal studies with longer follow-up periods are likely to yield more informative findings. Through serial testing in clinical practice, we currently provide evidence of the lack of association between vitamin D and lipids. Contrary to the cross-sectional association between Vitamin D levels and healthier lipid profiles, increasing Vitamin D levels from deficiency to an optimal range in a group neither improves nor worsens lipid profiles. This suggests that higher levels of Vitamin D may simply serve as a passive marker for better cardiovascular health.

## Conclusion

The results of this study demonstrate that there is no significant relationship between serum 25-hydroxyvitamin D levels and triglycerides and total cholesterol. However, there is a decrease in LDL levels with increasing serum 25-hydroxyvitamin D levels. Although this study was conducted on a large sample size, further evidence is needed to substantiate this theory. It is necessary to conduct similar research in other groups of individuals, such as autoimmune, metabolic, cardiovascular, and infectious disease patients, especially those with viral infections, to provide additional support for the hypothesis.

## Data Availability

The raw data required to reproduce these findings are available from the corresponding author upon request.
